# Methylation of promoter of RBL1 enhances the radioresistance of three dimensional cultured carcinoma cells

**DOI:** 10.18632/oncotarget.12647

**Published:** 2016-10-13

**Authors:** Dong Pan, Yaxiong Chen, Yarong Du, Zhenxin Ren, Xiaoman Li, Burong Hu

**Affiliations:** ^1^ Key Laboratory of Heavy Ion Radiation Biology and Medicine of Chinese Academy of Sciences & Key Laboratory of Space Radiobiology of Gansu Province, Institute of Modern Physics, Chinese Academy of Sciences, Lanzhou, China; ^2^ University of Chinese Academy of Sciences, Beijing, China; ^3^ Collaborative Innovation Center of Radiation Medicine of Jiangsu Higher Education Institutions and School for Radiological and Interdisciplinary Sciences (RAD-X), Soochow University, Suzhou, Jiangsu, China

**Keywords:** three dimensional cultured cells, radioresistance, methylation, promoter, RBL1

## Abstract

Three dimensional (3D) culture *in vitro* is a new cell culture model that more closely mimics the physiology features of the *in vivo* environment and is being used widely in the field of medical and biological research. It has been demonstrated that cancer cells cultured in 3D matrices are more radioresistant compared with cells in monolayer (2D). However, the mechanisms causing this difference remain largely unclear. Here we found that the cell cycle distribution and expression of cell cycle regulation genes in 3D A549 cells are different from the 2D. The higher levels of the promotor methylation of cell cycle regulation genes such as RBL1 were observed in 3D A549 cells compared with cells in 2D. The treatments of irradiation or 5-Aza-CdR activated the demethylation of RBL1 promotor and resulted in the increased expression of RBL1 only in 3D A549 cells. Inhibition of RBL1 enhanced the radioresistance and decreased the G2/M phase arrest induced by irradiation in 2D A549 and MCF7 cells. Overexpression of RBL1 sensitized 3D cultured A549 and MCF7 cells to irradiation. Taken together, to our knowledge, it is the first time to revealthat the low expression of RBL1 due to itself promotor methylation in 3D cells enhances the radioresistance. Our finding sheds a new light on understanding the features of the 3D cultured cell model and its application in basic research into cancer radiotherapy and medcine development.

## INTRODUCTION

Two dimensional (monolayer, 2D) cell culture is a traditional culture model which is widely used in the biological and medicinal studies. Whereas, big differences between the system of 2D cell culture and the physiological environment *in vivo* have been noted for very early. The morphology of cells *in vivo* are three dimensional (3D) due to a tightly interplay between the cell and its extracellular matrix (ECM) focal adhesions, as well as the actin cytoskeleton [1]. Meanwhile, cells *in vivo* interact with the peripheral environment in a three dimensional manner. The mechanical forces from the ECM and soluble chemicals around the environment affect the 3D cells’ behavior. In contrast, cells cultured in a monolayer such as the petri dish substrates do not have the environment such as ECM and therefore result in the difference considerably from 3D cells in their morphology and cell-cell and matrix-cell interactions [2–4]. Thus, the 2D cells can not show the actual physiological microenvironments *in vivo*. Animal models for studies *in vivo* are costly and complex. They also have problems of unpredictable propertis and ethical approval. It is an obvious and wise choice to use human cells to create a 3D model, which may utmost reproduce the physiological microenvrioments in human body [5]. 3D culture bridge the gap between the traditional cell culture and animal models [6, 7].

Matrigel basement membrane matrix is a commercial cell culture medium (BD Biosciences). It consist of a gelatinous protein mixture secreted by Engelbreth-Holm-Swarm (EHS) mouse sarcoma cells. The ECM components are rich in Matrigel and it was used commonly for 3D cell culture [8]. Compared to the traditional 2D culture, cells cultured in Matrigel demonstrate various differences in survival, proliferation, metabolism, differentiation, genes and proteins expression [9, 10]. In addition, the response behaviors of 2D and 3D cultured cells for stresses are also different [11, 12]. 3D cultured cancer cells are more chemo-resistant and radioresistant compared to 2D cells [13–16]. Our previous study showed that the 3D growth microenvironment in Matrigel impact on the reprogramming of differentiated cancer cells, which may in turn increase the radioresistance [17]. However, the reason behind the difference of radioresistance between 2D and 3D grown cancer cells remains largely unclear.

Epigenetic alterations are a kind of heritable changes in gene transcription or expression mode by regulating genome's structure and function, while the DNA sequence itself do not change [18]. It includes a series of molecular modifications including chromatin remodelling, DNA methylation, histone modifications, genetic imprinting, X chromosome inactivation and noncoding RNA (LncRNA, miRNA and siRNA, etc) regulated gene expression [19]. DNA methylation is an important epigenetic modifications of the genome. It involves in the regulation of many cellular processes via gene silencing without alteration in DNA sequences [20]. DNA methylation refers to the adding of a methyl group (−CH3) to the carbon 5 position of cytosine ring in a CpG dinucleotide by DNA methyltransferase (DNMTs) [21]. The remaining CpG dinucleotides are often methylated in the mammalian genome. Especially they are concentrated in CpG islands located in the upstream area of many genes from the transcriptional start site (promoter). Promoter regions of many tumor suppressor genes are hypomethylated, which allow their expression and maintain the normal state of the cell [22]. Thus, DNA methylation is an important mechanism resulting in the inactivation of protein-coding or non-coding genes in human cancers.

Accumulating evidence demonstrated that changes in methylation patterns resulted in the sensitivity or resistance of cancer cells to irradiation [23–29]. Kim *et al*. reported that CpG islands in promoter region of radioresistance related genes including PLXDC2, TOPO2A, GFI1, ETNK2 and IL12B were dramatically altered in the laryngeal cancer cells which are radioresistant [30]. Furthermore, breast cancer cells irradiated by the fractionated dose show specific DNA methylation alterations in several locus (TRAPP9, LINE1 and FOXC1), which are mainly the loss of methylation [31]. These studies suggest a connection among the radiation exposure, epigenetic modulation and carcinogenesis.

Whether is there difference in DNA methylation status between 2D and 3D cells, and these difference result in the higher cellular radioresistance in 3D than 2D cells? In current study, we found that the methylation of global DNA decreased gradually after irradiation, and the methylation of the promoter of RBL1 gene may play an important role in the induction of the radioresistance for 3D cells.

## RESULT

### Different morphological features and physiological characteristics between 3D and 2D cultured cells

**Figure 1:**
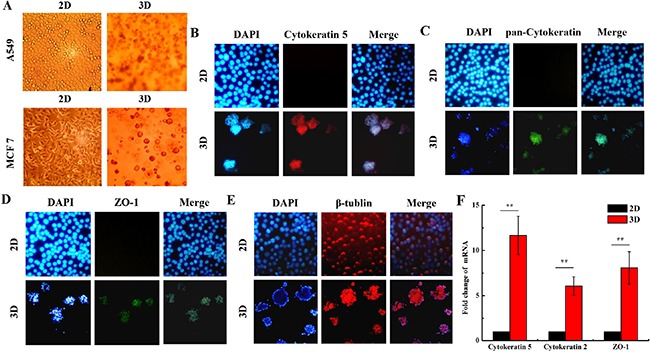
Different morphological features and biomarker expressions of organotypic tissue between 2D and 3D cultured cells The morphology of 2D and 3D cultured A549 and MCF7 cells were captured under phase-contrast microscope **A**. The 2D and 3D cultured A549 cells were immunostained with anti-Cytokeratin 5 **B**. anti-pan-Cytokeratin **C**.,anti-ZO-1 **D**. and anti-β-tublin **E**. and the representative confocal images are shown. **F**. Fold changes ofCytokeratin 2, 5 and ZO-1 mRNAs in 2D and 3D A549 cells. Each data point represents the mean of three independent experiments. Bars are the standard errors. Significance was determined by Student's t-test. **, P < 0.01.

Figure 1 shows the morphological features and some protein biomarkers expressed in 3D- and 2D-cultured A549 and MCF7 cells. 2D cultured cells are flat and a monolayer on the petri dish, while 3D cells cultured within the Matrigel form microspheres (Figure 1A). Cytokeratins family are proteins of the intermediary filaments of keratin, which are present in the cytoskeleton of all epithelial cells [32]. Monoclonal anti-pan-Cytokeratin may recognize epitopes presenting in many human epithelial tissues [32]. The expression of Cytokeratin 5 and pan-Cytokeratin were obviously observed in the 3D A549 cells, while they were very lower in the 2D (Figure 1B and Figure 1C). Zonula occludens protein-1 (ZO-1) is a member of membrane-related guanylate kinase family of proteins. It acts as a scaffold for organization of transmembrane tight junction proteins and enlists a variety of signaling molecules to tight junction [33]. We found that ZO-1 expressed in 3D cells. Inversely, it was negative for ZO-1 expression in the 2D cells (Figure 1D). β-tublin expressed in both 2D and 3D cells, which is the control for the immunostaining detection (Figure 1E). Accordingly, our qRT-PCR assay also indicated that the mRNAs of Cytokeratin 2, 5 and ZO-1 were higher in 3D A549 cells than in 2D (Figure 1F). These results indicate that 3D culture model is very close to the cells’ normal physiological microenvironment *in vivo*.

### The 3D cells are radioresistant

**Figure 2:**
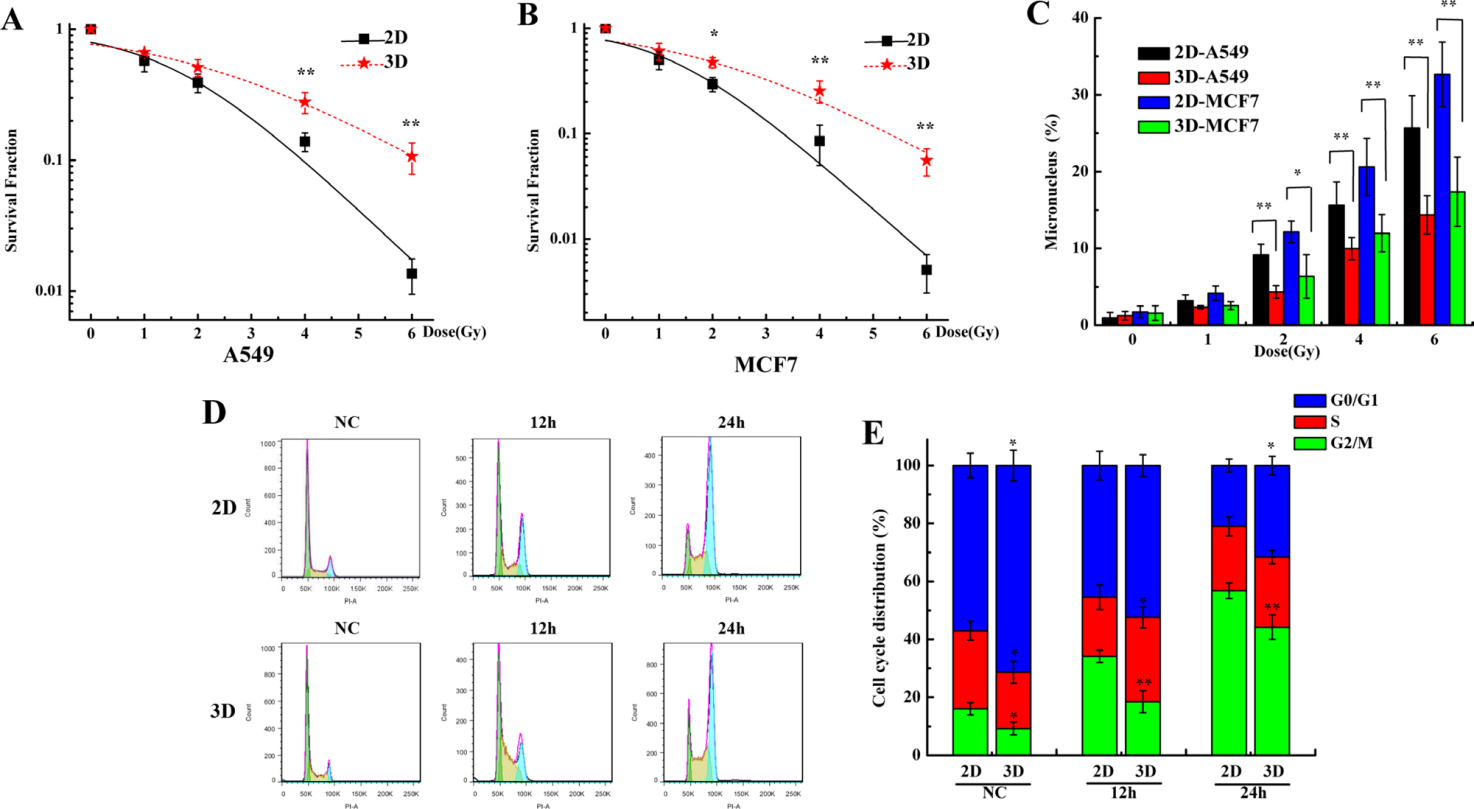
The 3D cultured cells are radioresistant compared with 2D cultured cells **A&B**. Survival fraction of A549 and MCF7 cells cultured in 2D and 3D after exposed to 0, 1, 2, 4 and 6 Gy X-rays and measured by colony formation assay. **C**. Micronucleus frequency of A549 and MCF7 cells cultured in 2D and 3D after exposed to 0, 1, 2, 4 and 6 Gy X-rays. **D&E**. Cell cycle distribution of 2D and 3D A549 cells after exposure to 4 Gy X-rays. Each data point represents the mean of three independent experiments. Bars are the standard errors. Significance was determined by Student's t-test. *, *P* < 0.05; **, *P* < 0.01.

The reports have shown that the 3D cells are radioresistant compared to the 2D cells [13–16]. It was confirmed again in our experimental system. Figure 2A and 2B show that the survival fractions of the 3D cultured A549 and MCF7 cells were more than those of the 2D cultured cells after exposed to X-rays. The micronucleus frequency in 3D cultured A549 and MCF7 cells after irradiation were lower compared with the 2D cells (Figure 2C). Figure 2D and 2E show that more 3D cultured A549 cells remained in G0/G1 phase and were lower in G2/M compared to the 2D both in unirradiated and irradiated groups. These results suggest that 3D cultured A549 and MCF7 cells are more radioresistant compared to the 2D cells.

### Low expression of RBL1, CCND1 and CCNF genes in 3D A549 cells

**Figure 3:**
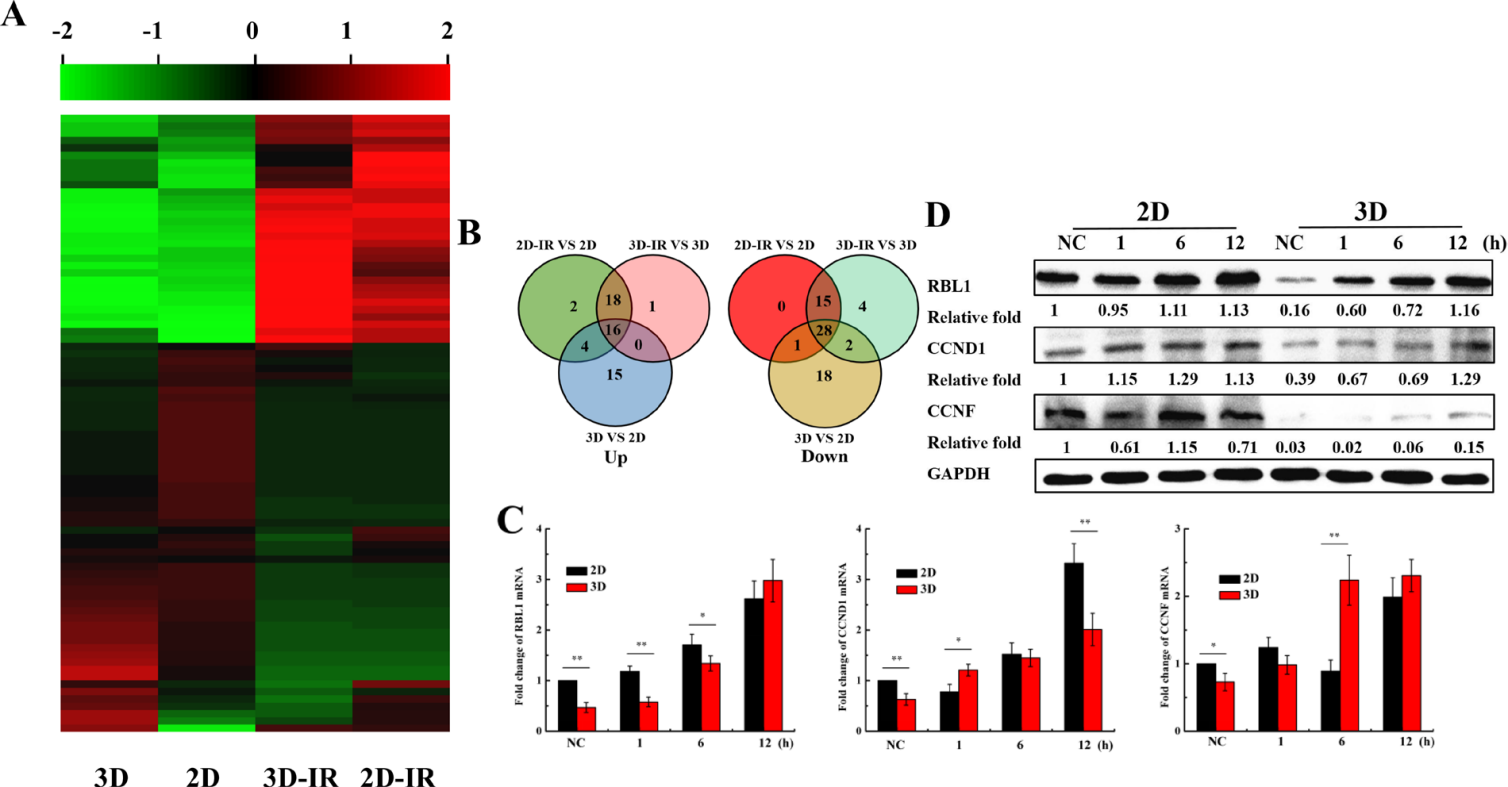
Expression of cell cycle regulation genes in irradiated 2D and 3D A549 cells **A**. Microarray analysis of 84 cell cycle regulation genes expression profile in 2D and 3D A549 cells 12h after irradiation with 4Gy X-rays. **B**. Venn diagrams of different expression of 84 cell cycle genes. **C**. Relative expression levels of RBL1, CCND1 and CCNF were measured by qRT-PCR at the indicated time points after 4 Gy X-rays in 2D and 3D A549 cells. GAPDH were used as internal control. **D**. The expressions of RBL1, CCND1 and CCNF at the indicated time points after 4 Gy X-rays in 2D and 3D A549 cells by western blot assay. Each data point represents the mean of three separate experiments. Bars are the standard errors. Significance was determined by Student's t-test. *, *P* < 0.05; **, *P* < 0.01.

Since the cell cycle distribution is different between 3D and 2D cultured A549 cells, we speculate that cell cycle regulation genes may play roles in the induction of the radioresistance for 3D cells. We thus tested the expression of cell cycle related genes for 3D and 2D A549 cultured cells exposed to irradiation. Figure 3A and 3B show that the expressions of some cell cycle regulation genes are different between 2D and 3D cells, especially, the Retinoblastoma-Like 1 (RBL1), Cyclin D1 (CCND1) and Cyclin F (CCNF) (Supplementary Table S1). Further, we verified the expression of RBL1, CCND1 and CCNF by qRT-PCR and western blotting in 3D and 2D cells at the time point 1, 6, 12 h after 4Gy X-ray irradiation. Figure 3C and 3D shows that the expression of above three genes were lower in unirradiated 3D-grown cells than in those of 2D. The expression of the three genes increased both in 3D and 2D A549 cells after irradiation, especially for RBL1 gene. These results indicate that RBL1, CCND1 and CCNF may be the underlying cause of higher radioresistance for 3D cultured cells.

### Hypermethylation in the promoters of 12 cell cycle regulation genes in 3D A549 cells

**Figure 4:**
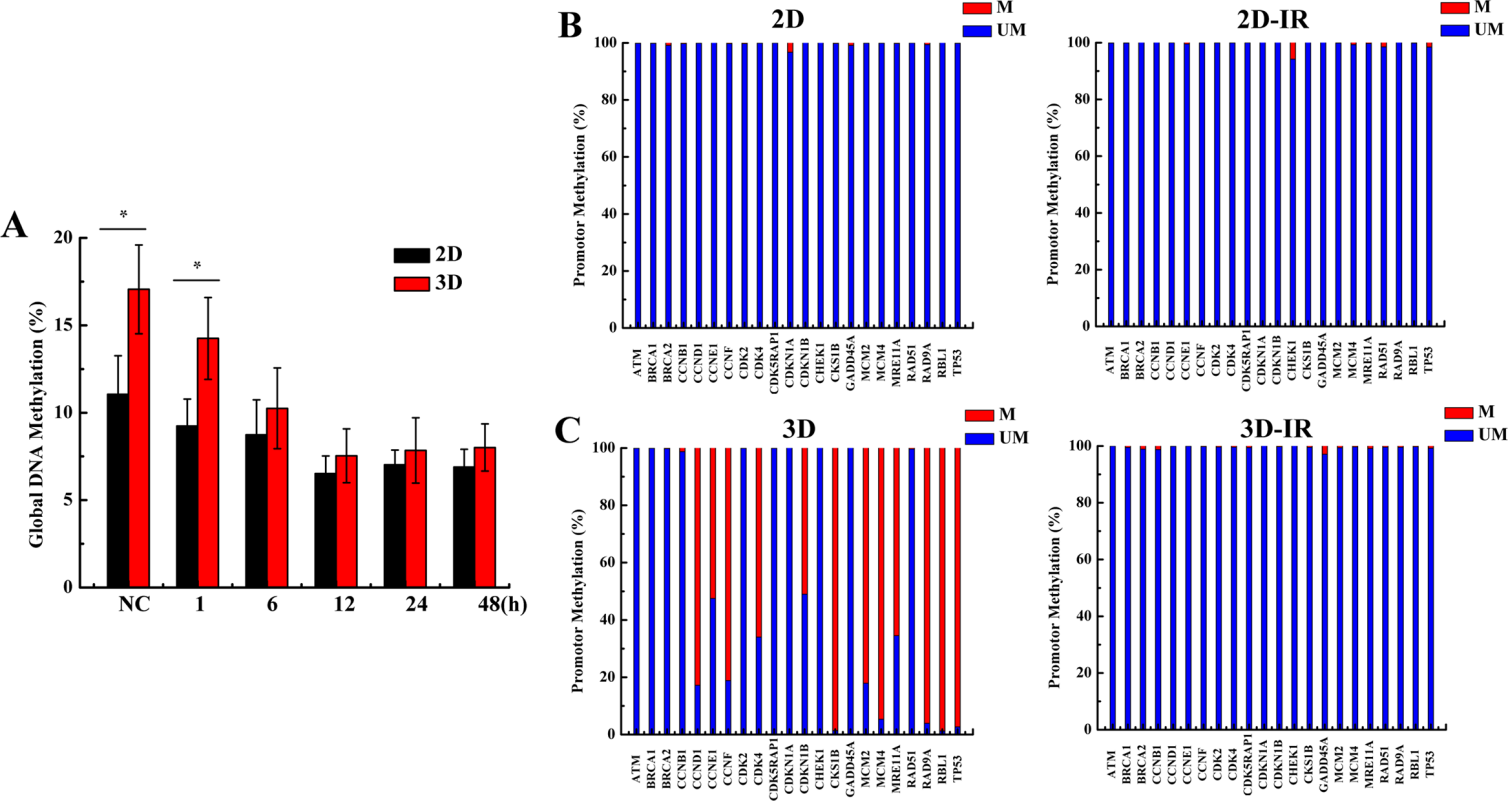
Methylation of genome and cell cycle regulation genes promotor status in irradiated 2D and 3D A549 cells **A**. Global DNA methylation status at the indicated 12h time points after 4 Gy X-rays irradiation in 2D and 3D cells. **B&C**. Methylation PCR array of 22 cell cycle genes promotor at 12 h after irradiation with 4 Gy X-rays in 2D and 3D cells. Each data point represents the mean of three separate experiments. Bars are the standard errors. Significance was determined by Student's t-test. *, *P* < 0.05; **, *P* < 0.01.

2D and 3D cultured A549 cells have same genetic background. What factor leads to the differences of cell cycle gene expression between 2D and 3D cells? DNA methylation is one of the epigenetic modifications which can regulate gene expression without altering DNA sequences [20, 21]. We speculate that DNA methylation is the potential reason for the differential expression of some cell cycle regulation genes between 3D and 2D cultured cells. We explored the global DNA methylation status in irradiated 3D and 2D cells with 4 Gy X-rays. As shown in Figure 4A, the DNA methylation level of 3D cells was slightly higher than 2D cells, and 4 Gy X-ray irradiation induced the decrease of DNA methylation both in 2D and 3D cells. Figure 4B and 4C shows that the promoters of 12 cell cycle regulation genes (CCND1, CCNE1, CCNF, CDK4, CDKN1B, CKS1B, MCM2, MCM4, MRE11A, RAD9A, RBL1, TP53) were hypermethylated and irradiation induced the demethylation at 12 h time point after exposed to 4Gy X-ray in 3D A549 cells. Conversely, all 22 cell cycle regulation genes promotor were hypormethylated in 2D cells and did not respond to irradiation.

### Demethylation of RBL1 promotor incuced by X-rays or 5-Aza-CdR and upregulation of RBL1 in 3D A549 cells

**Figure 5:**
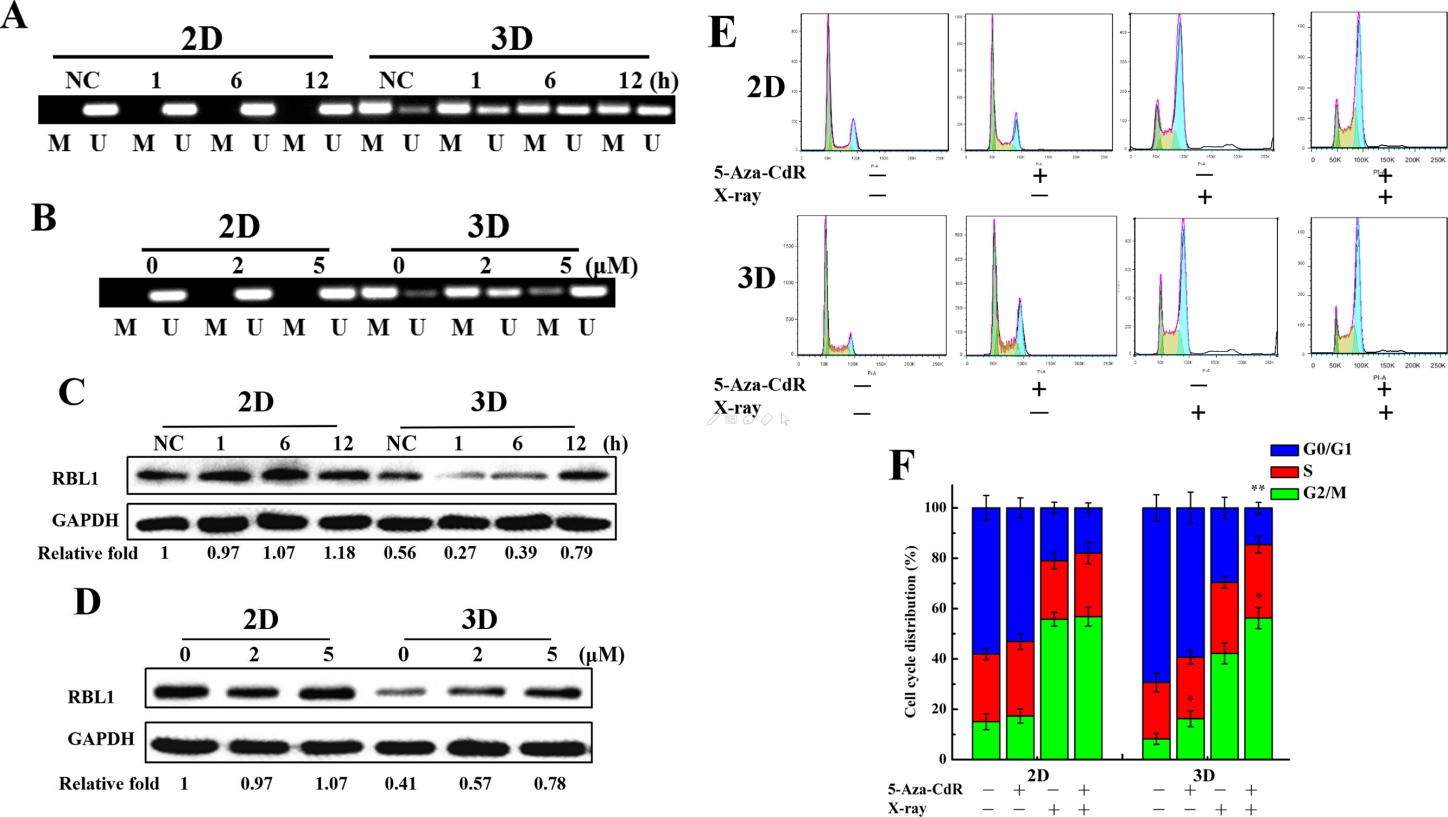
Methylation status of RBL1 promotor after X-rays or 5-Aza-CdR treatment and RBL1 expression in 3D A549 cells **A**. The changes of promoter methylation status of RBL1 at indicated time points measured by MSP in 4 Gy X-ray irradiated 2D and 3D cells. **B**. The changes of promoter methylation status of RBL1 measured by MSP in 5-Aza-CdR treated 2D and 3D cells. **C**. The expression of RBL1 in 2D and 3D cells at indicated time points after irradiation with 4 Gy X-rays by Western blot assay. **D**. The expression of RBL1 in 5-Aza-CdR treated 2D and 3D A549 cells by Western blot assay. **E&F**. Cell cycle distribution of 2D and 3D cells after 4 Gy X-ray irradiation combined with 2 μM 5-Aza-CdR treatment. Each data point represents the mean of three separate experiments. Bars are the standard errors. Significance was determined by Student's t-test. *, *P* < 0.05; **, *P* < 0.01.

As shown in Figure 3C, the expression of RBL1 was lower in 3D cells than 2D and increased most obviously in irradiated 3D cells, therefore, we only explored the methylation regulation of RBL1 in subsequent research. RBL1 is a tumor suppressor that has the same function as the retinoblastoma 1 (RB1) gene, which involves in cell cycle regulation [34]. In addition, it also participates in the cell radiation response [35]. However, the effect of the promotor methylation regulation of RBL1 on the biological function has not been reported yet. 5-aza-2’-deoxycytidine (5-Aza-CdR) is an inhibitor of DNA methyltransferase. The genomic hypomethylation induced by 5-Aza-CdR can lead to an increase of radiation sensitivity in cancer cells [36, 37]. Our previous study demonstrated that the 5-Aza-CdR treatment could decrease the global DNA methylation both in 3D and 2D cells, and effectively enhanced the radiosensitivity of 3D A549 cells [38]. We chose the concentration of 2 and 5 μM 5-Aza-CdR to treat for 72 h, which are relatively lower toxic, as the subsequent treatment. We sequenced the methylation status of RBL1 gene promoter in 2D and 3D cells after irradiation or 5-Aza-CdR treatment by MSP technique. As shown in Figure 5A, the upstream promoter region of RBL1 was methylated in unirradiated 3D cells, and then was demethylated gradually after 4Gy X-ray irradiation. In contrast, the upstream promoter region of RBL1 gene maintained the demethylated status both in unirradiated and irradiated 2D cells. 5-Aza-CdR also induced the demethylation of RBL1 promoter region in 3D cells (Figure 5B), which means that there is a dynamic change in the methylation status of RBL1 promoter region only in 3D cells. Further, we confirmed that the RBL1 protein level was higher in unirradiated 2D cells compared with the unirradiated 3D cells. The protein level increased 12 h after exposed to 4 Gy X-rays both in 2D and 3D cells (Figure 5C). In addition, 5-Aza-CdR treatment increased significantly the RBL1 protein level in 3D cells while it was not affected in 2D cells (Figure 5D). These results suggest that the methylation modulation occurred in RBL1 promotor region after X-rays or 5-Aza-CdR treatment and upregulated the expression of RBL1 in 3D cells. Our previous results demonstrated that there were more G0/G1 phase cells in 3D A549 cell population and irradiation induced more G2/M phase arrest in 2D A549 cells (Figure 2C and 2D), thus we wonder whether methylation status affect the cell cycle distribution both in 2D and 3D cells. As shown in Figure 5E and 5F, 2 μM of 5-Aza-CdR treatment increased significantly the G2/M phase arrest in 3D while not in 2D cells, and aggravated the G2/M phase arrest in irradiated 3D A549 cells. These data indicate that the difference in the methylation status of cell cycle related genes between 2D and 3D A549 cells account for the differential cell cycle distribution.

### Knockdown of RBL1 enhances the radioresistance of 2D cultured cancer cells and decreases the G2/M phase arrest induced by X-rays

**Figure 6:**
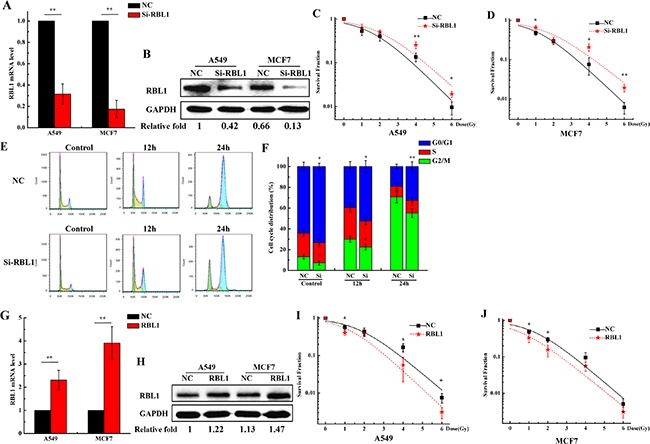
Knockdown of RBL1 enhances the radioresistance of 2D cultured A549 and MCF7 cells and decreases the G2/M arrest induced by X-ray **A**. Relative expression levels of RBL1 mRNA were measured by qRT-PCR after transfected with RBL1-siRNA or negative control (NC) in 2D A549 and MCF7 cells. GAPDH were used as internal control. **B**. Western blot assay of RBL1 protein level after being transfected with RBL1-siRNA or NC into 2D A549 and MCF7 cells. **C&D**. Survival fractions of 2D A549 and MCF7 cells transfected with RBL1-siRNA or NC, was measured by colony formation assay after exposed to 0, 1, 2, 4 and 6 Gy X-rays. **E&F**. Cell cycle distribution of 2D A549 cells transfected with RBL1-siRNA or NC and then exposed 4 Gy X-rays. **G**. Relative expression levels of RBL1 mRNA were measured by qRT-PCR after being transfected with RBL1 overexpression vector or negative vector (NC) in 2D A549 and MCF7 cells. **H**. Western blot assay of RBL1 protein level after being transfected with RBL1 overexpression vector or NC into 2D A549 and MCF7 cells. **I&J**. Survival fractions of 2D A549 and MCF7 cells transfected with RBL1 overexpression vector or NC were measured by colony formation assay after exposed to 0, 1, 2, 4 and 6 Gy X-rays. Each data point represents the mean of three separate experiments. Bars are the standard errors. Significance was determined by Student's t-test. *, *P* < 0.05; **, *P* < 0.01.

It has been reported that RBL1 involves in the cell radiation response [35]. Since the expression of RBL1 protein was regulated depending on the promotor methylation and was lower in 3D cells when compared with 2D cells, we speculate that RBL1 may play an important role in the radioresistance induction for 3D cultured carcinoma cells. To validate our assumption, we knocked down the expression of RBL1 to investigate the radiosensitivity of 2D A549 cells. As shown in Figure 6A and 6B, both mRNA expression and protein level of RBL1 significantly decreased at 48 h after being transfected with the siRNA of RBL1, compared with the transfected siRNA-negative control (NC) in 2D cultured A549 and MCF7 cells. It means that the selected siRNA of RBL1 worked perfectly. Figure 6C and 6D show that the survival fractions of X-ray irradiated 2D A549 and MCF7 cells transfected with RBL1-siRNA were higher than those of negative control, suggesting that the knockdown of RBL1 enhances the radioresistance of 2D cultured cells. In addition, inhibition of RBL1 induced more 2D A549 cells remaining in G0/G1 phase, and the G2/M phase arrest decreased significantly at the time point of 12 and 24 h after the irradiation with 4 Gy X-ray (Figure 6E and 6F), compared with the NC groups. Meanwhile, we also investigated whether the radiosensitivity of 2D A549 cells increased when the RBL1 level were upregulated in 2D cells. Figure 6G and 6H show both mRNA expression and protein level of RBL1 significantly increased at 48 h after being transfected with the RBL1 overexpression vector, compared with the transfected negative vector in 2D cultured A549 and MCF7 cells. Accordingly, the survival fractions of X-ray irradiated 2D A549 and MCF7 cells transfected with RBL1 overexpression vector were lower than those of negative control (Figure 6I and 6J), suggesting that the overexpression of RBL1 weakens the radioresistance of 2D cultured cells.

### Overexpression of RBL1 sensitizes the 3D cultured A549 and MCF7 cells to X-rays

**Figure 7:**
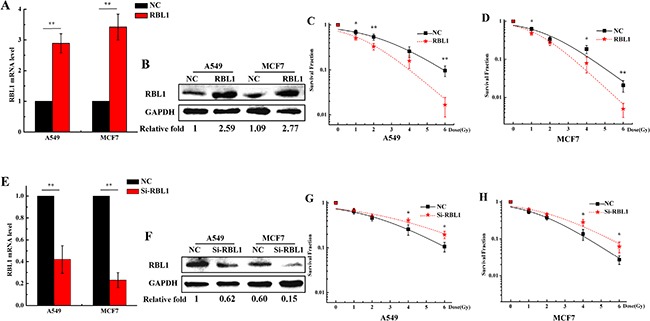
Overexpression of RBL1 sensitizes the 3D cultured A549 and MCF7 cells to X-rays **A**. Relative expression levels of RBL1 mRNA were measured by qRT-PCR after being transfected with RBL1 overexpression vector or negative vector (NC) in 3D A549 and MCF7 cells. GAPDH were used as internal control. **B**. Western blot assay of RBL1 protein level after being transfected with RBL1 overexpression vector or NC into 3D A549 and MCF7 cells. **C&D**. Survival fractions of 3D A549 and MCF7 cells transfected with RBL1 overexpression vector or NC was measured by colony formation assay after exposed to 0, 1, 2, 4 and 6 Gy X-rays. **E**. Relative expression levels of RBL1 mRNA were measured by qRT-PCR after transfected with RBL1-siRNA or negative control (NC) in 3D A549 and MCF7 cells. **F**. Western blot assay of RBL1 protein level after being transfected with RBL1-siRNA or NC into 3D A549 and MCF7 cells. **G&H**. Survival fractions of 3D A549 and MCF7 cells transfected with RBL1-siRNA or NC, were measured by colony formation assay after exposed to 0, 1, 2, 4 and 6 Gy X-rays. Each data point represents the mean of three independent experiments. Bars are the standard errors. Significance was determined by Student's t-test. *, *P* < 0.05; **, *P* < 0.01.

Since knockdown of RBL1 enhanced the radioresistance of 2D cultured A549 and MCF7 cells (Figure 6C), whether does the overexpression of RBL1 increase the cellular radiosensitivity in 3D cells? Firstly, we transfected the RBL1 overexpression vector and negative vector to 2D A549 and MCF7 cells. After the transfection of vectors, the positive clones were selected by G418 and the stable highly expressing RBL1's cell line were cultured in 3D. As shown in Figure 7, both mRNA expression (Figure 7A) and protein level (Figure 7B) of RBL1 significantly increased in 3D cultured A549 and MCF7 cells which contain the overexpression vector of RBL1, compared with the negative groups. Figure 7C and 7D show that the survival fractions of RBL1 overexpressed 3D A549 and MCF7 cells exposed to X-rays were higher than those of the negative control, which suggests that the overexpression of RBL1 sensitizes 3D cultured A549 and MCF7 cells to X-rays. Further, we knocked down the expression of RBL1 of 3D cultured A549 (Figure 7E) and MCF7 (Figure 7F) cells through transfection with the siRNA of RBL1. The corresponding survival fractions of X-ray irradiated 3D cells transfected with RBL1-siRNA were higher than those of the negative control (Figure 7G and 7H), suggesting that low expression of RBL1 cause the radioresistance of 3D cells, compared to the 2D cells.

## DISCUSSION

Lung cancer is the second common cancer worldwide [39]. It is estimated that lung cancer accounts for 1.6 million newly registered cases of cancer and for 1.37 million cancer deaths annually [40]. Breast cancer is the most invasive form of cancer in women [41], and the incidence of the disease is rising in many countries [42]. Meanwhile, radiotherapy is a major method in the curative treatment for mostly solid cancer, therefor, exploring the molecular mechanisms of radioresistant in tumor cells is a common emphasis in the application of clinical radiotherapy [43]. Therefore, A549 and MCF7 cells were exploited in our subsequent experiments to investigate the radiosensitivity in 3D model for developing the effective radiotherapy strategy.

The traditional 2D cell culture overlooks the interplay between cells and their microenvironment. Cells cultured in Matrigel exhibit a round morphology which are in three dimensional, such as the shown in Figure 1A. Meanwhile, 3D cell cultured model can simulates the physiology features *in vivo* and is being used widely in the field of medical and biological research [7, 17, 44]. Our data also showed that the expressions of Cytokeratins and ZO-1, which are tight junction proteins, were observed only in 3D A549 cells. In addition, it has been demonstrated that 3D-grown cancer cells are chemoresistant and radioresistant compared to the 2D cells [11–17]. Our results are also consistent with their findings (Figure 2A and 2B). Furthermore, we found that more 3D A549 cells remained in G0/G1 phase and lower in G2/M phase compared with 2D cells both in unirradiated and irradiated groups. Thus, we speculated that cell cycle relative genes may play crucial role for the radioresistance of 3D cultured cells. Our results showed that the expression of some cell cycle regulation genes were different between 2D and 3D cultured A549 cells, especially the RBL1, CCND1 and CCNF (Figure 3C, 3D and supplementary Tables S1). Combined with our further test, we found the different expression of RBL1, CCND1 and CCNF between 2D and 3D cells. These results suggest that differently expressed RBL1, CCND1 and CCNF may increase the radioresistance of 3D cultured cells.

Although 3D cells have the same genetic background as the 2D cells, the expressions of cell cycle genes are different between 2D and 3D cells. Epigenetic alterations, especially DNA methylation modification, regulates genes expression and involves in the regulation of various cellular biological processes without alteration in DNA sequences [18–21]. Thus, we considered that the DNA methylation status is the potential reason in the induction of the high radioresistance for 3D cultured cells. There are four types of DNA methyltransferase (DNMT) in mammals: DNMT1, DNMT2, DNMT3a and DNMT3b [45, 46]. Changes in DNMTs and genomic DNA methylation after irradiation have been indicated [25, 45–49]. Similarly, our results demonstrated that the DNA methylation level of 3D cells was slightly more than that of 2D cells and decreased gradually after irradiation both in 3D and 2D cells. Moreover, 12 cell cycle regulation genes promotor are hypermethylated and irradiation induced the demethylation in 3D cells. However, all 22 genes’ promotor are hypormethylated and do not respond to radiation for 2D cells. Lower expression levels of DNA methyltransferases such as DNMT1 usually parallel with the hypomethylation in cells [25–29]. We found that the DNMT1 level was higher in 3D A549 cells than in 2D cells and decreased gradually after irradiation exposure both in 3D and 2D A549 cells (Supplemental Figure S1A). These results suggest that methylation status of promotor account for the 12 cell cycle regulation genes low expression in 3D A549 cells and DNMT1 may involve in this process.

Although we found the differential expressions of CCND1 and CCNF between 3D and 2D cells post-irradiation, the changes of expression of CCND1 and CCNF were lower than the RBL1 in irradiated 3D cells at the indicated time point. Even their promotors also occur demethylation, the upregulations of the gene expressions were not significant (Figure 3C and 3D). Therefore, we considered that RBL1 could play important roles in the radioresistance induction in 3D cells through the mechanism of methylation regulation and we, thus, only explored the methylation regulation of RBL1 in subsequent research. RBL1 is a tumor suppressor gene that are thought to regulate cell cycle progression by directly inhibiting CDK2 activity and participate in the cell radiation response [34, 35, 50, 51]. Therefore, we explored the methylation status of RBL1 promotor region and the effect on radioresistance in the subsequent experiments. Our early study demonstrated 5-Aza-CdR effectively enhanced the radiosensitivity of 3D cells [38]. Here, our results demonstrated that the RBL1 promotor hypermethylation and the demethylation caused by irradiation or 5-Aza-CdR was only in 3D A549 cells, indicating that there is a dynmic methylated regulation in RBL1 promoter region of 3D cells after irradiation. Together with the RBL1 protein levels increase in 3D A549 cells after irradiation or 5-Aza-CdR treatment indicates that methylation modulation of RBL1 promotor in 3D A549 cells affect the expression of RBL1. DNA methylation status differences of cell cycle relative genes may account for the different cell cycle distribution in cells because our experiment showed that 5-Aza-CdR could significantly increase the G2/M phase in 3D A549 cells while not in 2D A549 cells, and aggravate G2/M phase arrest in irradiated 3D A549 cells. Interestingly, previous reports demonstrated that CCND1 regulate the activation of RBL1 in T98G cells [52]. In our current experiments the expression and promotor methylation status of CCND1 were different between 2D and 3D A549 cells, implying that CCND1 involves in the RBL1 regulation in 3D cells and the epigenetic regulations are collaborative and complicated. The low expression of CCND1 and CCNF may also result in the 3D cells radioresistance. However, we deem that methylation of the promoter in RBL1 gene result in the low expression of RBL1 protein and cause the radioresistance of 3D cells.

RBL1 can be phosphorylated during the transition of S to M phase and is dephosphorylated during the G1 phase, and protein phosphatase 2A (PP2A) is responsible for the progress [53–55]. RBL1 is capable to response to DNA damage agents such as irradiation or addition of cisplatin [33, 34]. Meanwhile PP2A radiosensitizes carcinoma cells by inducing mitotic catastrophe and blocking DNA damage repair [56]. Thus, we speculate that RBL1 involves in mediating the radioresistance of 3D cell. To validate our hypothesis, we investigated whether low expression of RBL1 enhanced the radioresistance in 2D A549 cells. Our data demonstrated that the survival fractions of RBL1 inhibited A549 and MCF7 cells were higher than the NC groups. In addition, compared to the NC groups, inhibition of RBL1 increased the fraction of G0/G1 phase and decreased significantly the arrest of G2/M phase. Because the expression of RBL1 in 3D cells is already lower, we did not investigate the effect of inhibition of RBL1 in 3D cells. Furthermore, overexpression of RBL1 sensitized the 3D cultured A549 and MCF7 cells to X-rays. The series of results suggest that low expression of RBL1 account for the radioresistance of 3D carcinoma cells.

Taken together, our results demonstrate that the hypermethylation of promotor of cell cycle relative genes in the 3D cultured cells leads to the differences of the cell cycle distribution and expression of cell cycle regulation genes between the 3D and 2D cells. The low expression of RBL1 caused by itself promotor methylation enhances the radioresistance of 3D cultured cells. Our study reveals the underlying mechanism of radioresistance of 3D cultured cancer cells and contributes to understand the features of 3D cultured cell model and its application in medicine basic research and cancer radiotherapy.

## MATERIALS AND METHODS

### Cell culture

A549 cells (human lung carcinoma cells) and MCF7 cells (human breast carcinoma cells) were obtained from the American Type Culture Collection (Manassas, VA, USA). For 2D-grown cultures, A549 cells were cultured in RPMI-1640 medium (Gibco, USA) supplemented with 10% FBS (Hyclone, USA) and 1% penicillin/streptomycin (Amresco, USA). MCF7 cells were cultured in Dulbecco’ Modified Eagle's Medium (DMEM) (Gibco, USA) supplemented with 10% FBS and 1% penicillin/streptomycin. For 3D-grown cultures, construction of the 3D growth microenvironment using Matrigel (BD, USA) was performed mainly as described previously [17, 57]. Briefly, 30 μL trypsinized at a density of 1.5 ×10^6^ cells/mL were mixed with 250 μL pre-thawed Matrigel and seeded into single wells of a 12-well tissue culture plate, and then incubated for 30 min at 37°C to allow the mixture to gel, added appropriate medium. All experiments with 3D-grown cells were cultured in Matrigel for 5d, changing medium every 2 days. Both 2D- and 3D-grown cells were cultured at 37°C in a humidified atmosphere containing 5% CO_2_.

### Immunostaining

The primary antibodies used for immunostaining of 2D and 3D cells include: ZO-1(1:500, Abcam, USA), Cytokeratin 5 (1:500, CST, USA), Pan-cytokeratin (1:500, Abcam) and β-tublin (1:1000, CST). Secondary antibodies (anti-mouse or rabbit conjugated with Alexa 488/633) were purchased from Beyotime (1:2000, China).

For 2D cultures, cells were seeded at 1×10^4^ cells in each well of 24-well tissue culture plate, cultured for 48 h prior to be detected. Cells were fixed with 4% paraformaldehyde (PFA) for 20 min at room temperature and permeabilized with 0.5% Triton X-100 in PBS while on ice for 10 min. Non-specific binding sites were blocked with 5% bovine serum albumin (BSA) in PBS for 60 min at room temperature prior to probing with primary antibodies. Then the cells were incubated in the primary antibody diluted in 5% BSA/1× PBS 1h at room temperature. After incubation, cells were washed three times with PBS, 10 min each and then incubated with the appropriate Alexa Fluor secondary antibodies diluted in 5% BSA for 1 h. Cells were washed three times with PBS for 10 min each again. The nuclei were counterstained with DAPI (Beyotime, China). The 3D structures in Matrigel were immunostained as described previously [57] with the following modifications: the 3D cultures were fixed with 4% PFA for 30 min at room temperature and thereafter washed three times with PBS for 20 min each. Blocking was done by incubating the structures in immunofluorescence (IF) buffer (5% BSA/0.2% Triton X-100 in PBS) for 4 h. The 3D structures were incubated in the primary antibody diluted in 5% BSA/1×PBS overnight at room temperature. After incubation, the structures were washed three times with PBS, 20 min each and then incubated with the appropriate Alexa Fluor secondary antibodies diluted in 5% BSA for 1 h. The structures were washed three times with PBS for 20 min each. The nuclei were counterstained with DAPI. Analyses were performed with a fluorescence microscope (Axio Imager Z2) at 20× magnification.

### Radiation

X-ray irradiation was carried out with a Faxitron RX-650 facility (Faxitron Bioptics, USA), which was operated at 100 kVp 5 mA at room temperature. The target of this instrument is wolframium (W). The dose rate was 0.92 Gy/min. The dose rate was measured using the Ray meter (RX-650, Germany).

### Conversion of 3D structure to monolayer

3D structures were recovered from Matrigel using Recovery Solution (BD, USA) according to the manufacturer's instructions as described previously [17, 58]. Briefly, 3D cultures were first washed with ice-cold PBS, and then the Matrigel containing the 3D structures was removed from the well, transferred to 15 mL tube containing the pre-chilled recovery solution (1 mL per well), incubated on ice for 45min with intermittent mixing and then centrifuged at 1000 rpm for 10 min at 4°C. The supernatant containing the dissolved Matrigel was discarded and the 3D structures were washed once with PBS. To make single-cell suspension of recovered 3D structures, cells were trypsinized using trypsin-EDTA (0.25%, Invitrogen).

### Clonogenic survival assay

For 2D culture, irradiated cells were washed with PBS buffer, trypsinized and counted using a cell counter (Coulter) after irradiation. An appropriate number of cells were plated into each 60-mm dish to produce colonies. For 3D culture, the irradiated and control cells were first recovered from Matrigel by using Recovery Solution (BD) on ice for 30 min, and then trypsinized and resuspended in medium. An appropriate number of cells were plated into each 60-mm dish to produce colonies. After incubating for 10 days, the cells were fixed with 10 mL fresh Carnoy's fluid, stained with 0.5% crystal violet for 20 min. The number of colonies with greater than 50 cells were counted as survivors. Plating efficiencies (PE) were calculated as follows: numbers of colonies formed/numbers of cells plated. Surviving fractions were calculated as follows: PE (irradiated)/PE (unirradiated). The parameters of the survival curve, such as the α and β values, were obtained from the survival fraction (SF) data by curve fitting using the nonlinear model as follows: SF = (1 + exp(−α*(D - β)))^−1^. Where D is the radiation dose delivered to the cells. All experiments were performed in triplicate. The experiment was at least repeated for three times independently.

### Micronucleus assay

For 2D cells, 48 h after radiation, cells were fixed with Carnoy's fluid for 20 min at room temperature, stained with 20 μL of Acridine Orange in an aqueous solution (10 μg/mL). For 3D cells, conversing the 3D structure to monolayer after irradiation and treated as the 2D cells on 48 h after radiation. Analyses were performed with a fluorescence microscope (Axio Imager Z2) at 20× magnification. At least 500 cells were scored for each sample. The experiment was at least repeated for three times independently.

### Flow cytometry analysis

For cell cycle analysis, 2D and 3D cells were harvested by trypsinization and recovery solution respectively, washed with PBS twice, fixed in 70% ethanol overnight at 4°C and then cells were pelleted and resuspended in PBS at 1 × 10^6^ cells/mL and incubated with 100 μg/mL DNase-free RNase A, 0.2% Triton X-100 and stained with 50 μg/mL PI (Sigma, USA) at 4°C for 30 min. A total of 10^4^ nuclei were examined in a BD LSRFortessa™ cell analyzer (BD Biosciences) and DNA histograms were analyzed by Flowjo software. The experiment was at least repeated for three times independently.

### RNA and DNA extraction

Total RNA was extracted from cultured cells by using TRIzol Reagent (Life Technologies) according to the instruction. Genomic DNA was extracted from the cells by using Wizaed^®^ SV Genomic DNA Purification System (Promega) according to the instruction.

### PCR array of human cell cycle genes

The RT^2^ Profiler PCR Array Kit (Qiagen, Germany) was used to profile the expression of 84 cell cycle regulation genes. After the genomic DNA was extracted, all steps were carried out according to the manufacturer's instruction. PCR was performed on samples using a Bio-Rad Chromo4 System RealTime PCR detector (Bio-Rad, USA). The relative fold change of mRNA was calculated by using the 2^−ΔΔ^Ct method following equation: RQ (Relative Quantitation) = 2^−ΔΔ^Ct.

### Global DNA methylation analysis

The global DNA methylation levels of extracted genomic DNA were detected by the Methylated DNA Quantification Kit (Epigentek, China) according to the manufacturer's instruction. Briefly, 200 ng global DNA of every sample was added in the well plate. After incubating the plate in 37°C for 30 min, the antibody was added in the wells and incubated for 1 h at room temperature. Then the second antibody was added and incubated for 1 h at room temperature. The substrate was added and shielded from light for 15 min before the assay was executed. According to the protocol, the absorbance was measured at 420 nm and determined with a microplate reader. Every experimental treatments were performed in triplicate wells. The experiment was at least repeated for three times independently.

### Cells treated by 5-Aza-CdR

According our previous research [38], 5-Aza-CdR (Sigma, USA) were dissolved in dimethylsulfoxide (DMSO), and mixed with fresh medium. Cells were treated 5-Aza-CdR in different concentration for 72 h before the subsequent experiments. Replaced the fresh medium with 5-Aza-CdR every 24 h. The negative control groups were added in same dosage of DMSO and replaced the fresh medium with DMSO every 24 h. The experiment was at least repeated for three times independently.

### Methylation PCR array

The EpiTect Methyl Signature PCR Array (Qiagen) Kit was used to profile the promoter methylation status of a panel of 22 cell cycle regulation genes. According to the manufacturer's instruction, DNA was treated with a simple restriction enzyme digestion and RT-PCR, results can show the promoter methylation status of 22 different cell cycle regulators with 2^−ΔΔ^Ct method. PCR was performed on samples using a Bio-Rad Chromo4 System RealTime PCR detector (Bio-Rad).

### Promoter methylation analysis

The extracted genomic DNA was subjected to bisulfate modification by using the EpiTect Fast DNA Bisulfite Kit (Qiagen) according to the manufacturer's instruction. After that the bisulfate converted genomic DNA was amplified by a set of primers for the unmethylated reaction and the methylated reaction to the methylation-specific PCR (MSP): unmethylated forward primer (5′ GGAGGTATTTTATTATGTTGTATGA) and reverse primer (5′ TCCTTAACCCTTAACTAATCACAAA), methylated forward primer (5′ GGAGGTATTTTA TTACGTTGTACGA) and reverse primer (5′ CTTAAC CCTTAACTAATCGCGAA). PCR was carried out with MyCycler RCR (Bio-Rad) by using the following condition: 94°C for 5 min, followed by 40 cycles of 94°C for 30 s, 52°C (unmethylated primer) or 56°C (methylated primer) for 30 s, and 72°C for 30 s.

### Quantitative real-time polymerase chain reaction (qRT-PCR) for mRNA expression

For mRNA detection, reverse transcription was carried out with Transcriptor First Strand cDNA Synthesis Kit (Roche, Switzerland), and qRT-PCR was carried out with SYBR Green PCR Master (Roche). The primers of Cytokeratin 2, Cytokeratin 5, ZO-1, RBL1, CCND1, CCNF and internal control of GAPDH were purchased from GeneCopoeia (Guangzhou, China). qRT-PCR was performed on samples using a Bio-Rad Chromo4 System Real Time PCR detector (Bio-Rad). All steps were carried out according to the protocol. The relative fold change of mRNA was calculated by using the 2^−ΔΔ^Ct method. The experiment was at least repeated for three times independently.

### Western blot

Cells were lysed in RIPA buffer (Beyotime, Shanghai, China) with Protease Inhibitor Cocktail Tablets (Roche, Switzerland). The total protein concentrations of the lysates were determined using a protein assay kit (Bio-Rad, USA). Equal amounts of protein were denatured with loading buffer (Beyotime, Shanghai, China) at 100°C for 10 min, then loaded in 12% SDS-PAGE for electrophoresis, and transferred to a methanol-activated polyvinylidene fluoride membrane (Millipore, USA). The membrane was blocked in tris-buffered saline (TBS) containing 5% bovine serum albumin (MP Biomedical, USA) for 2h at room temperature. Primary antibodies were incubated overnight at 4°C. The primary antibodies include: RBL1 (1:1000, Proteintech, China), CCND1, CCNF (1:1000, Affinity Biosciences, USA) and GAPDH (1:1000, ZSGB-BIO, Beijing, China). After washing with TBS, the membrane was incubated with the appropriate horseradish peroxidase (HRP)-labeled secondary antibody for 1 h at room temperature. Secondary antibody conjugated with HRP is Goat-Anti Rabbit IgG (1:5000, ZSGB-BIO, Beijing, China). The relative protein level is quantified using Image J software.

### Cell transfection

SiRNA that targets to RBL1 and its negative control were purchased from RiboBio (Guangzhou, China). RBL1 siRNA (sense: 5′ GCCGGUUACAGAGUAUUGUTT-3′, antisense: 5′ ACAAUACUCUGUAACCGGCTT -3′) was constructed as described [59]. Plated the A549 cells and MCF7 cells on the day before transfection at a confluence of 30%−50%. Transfection was performed with Lipofectamine 2000 (Invitrogen, USA) according to the manufacturer's instruction. The medium was changed with new culture medium 6 h post-transfection. The cells used in the following experiments were transfected for 48 h. Plasmid for expression of human RBL1 and its negative control were purchased from GeneCopoeia (Guangzhou, China). After the transfection of plasmid, the positive clones were selected by G418 (500 μg/mL), and the stable highly expressing RBL1 cell line was harvested. The highly expressing RBL1 cells were seeded in Matrigel for 5d and used in the following experiments.

### Statistics

The statistical significance (*P* values) in mean values of two-sample comparison was determined with Student's t-test. A value of *P* < 0.05 was considered statistically significant (*) and a value of *P* < 0.01 was considered extremely significant (**). Values shown on graphs represent the means ± s.d of at least three independent repeated experiments.

## SUPPLEMENTARY FIGURE AND TABLE




