# Supplementary material for: Methylation of promoter of RBL1 enhances the radioresistance of three dimensional cultured carcinoma cells
Source: Oncotarget. 2016 Oct 13;8(3):4422–35. doi: 10.18632/oncotarget.12647 (PMC5354843; doi:10.18632/oncotarget.12647)
Supplement: Supplementary file 1 [file oncotarget-08-4422-s001.pdf]

Methylation of promoter of RBL1 enhances the radioresistance of three dimensional cultured carcinoma cells

SUPPLEMENTARY FIGURE AND TABLE

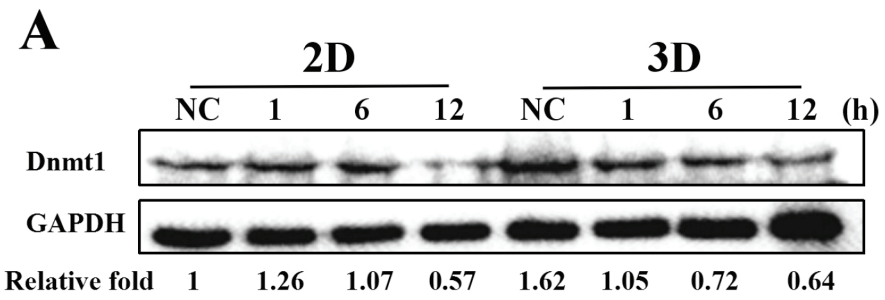

Supplementary Figure S1: (A) The expression level of DNMT1. The expression level of DNMT1 at indicated time points after irradiation with 4 Gy X-rays in 2D and 3D A549 cells by Western blot assay.

Supplementary Table S1: Up-Down Regulation (comparing to 2D as control group)

See Supplementary File 1
