# Supplementary material for: Methylation of promoter of RBL1 enhances the radioresistance of three dimensional cultured carcinoma cells
Source: Oncotarget. 2016 Oct 13;8(3):4422–35. doi: 10.18632/oncotarget.12647 (PMC5354843; doi:10.18632/oncotarget.12647)
Supplement: Supplementary file 2 [file oncotarget-08-4422-s002.docx]

| **Up-Down Regulation (comparing to 2D as control group)** | | | |
| --- | --- | --- | --- |
| **Gene Name** | **2D-IR** | **3D** | **3D-IR** |
|  | **Fold Change** | **Fold Change** | **Fold Change** |
| **ABL1** | **1.0928** | **1.1567** | **-1.2193** |
| **ANAPC2** | **1.7508** | **1.5369** | **-1.5433** |
| **ATM** | **-1.1188** | **-1.2397** | **1.4886** |
| **ATR** | **-1.1345** | **-2.5669** | **-1.4103** |
| **AURKA** | **-3.4153** | **-2.3134** | **-9.3567** |
| **AURKB** | **-1.1826** | **-1.6935** | **-1.9** |
| **BCCIP** | **1.1313** | **-2.639** | **-1.2623** |
| **BCL2** | **-1.0154** | **-2.2974** | **-4.9109** |
| **BIRC5** | **-1.9346** | **-3.4105** | **-3.0022** |
| **BRCA1** | **1.2995** | **-1.0943** | **-1.7243** |
| **BRCA2** | **1.3832** | **-2.1886** | **-3.2176** |
| **CASP3** | **-1.0958** | **-1.4948** | **-2.4897** |
| **CCNA2** | **-1.5284** | **-3.0951** | **-3.4967** |
| **CCNB1** | **-4.7966** | **-3.0105** | **-5.0141** |
| **CCNB2** | **-2.0028** | **-2.4116** | **-2.4385** |
| **CCNC** | **1.1471** | **-2.2038** | **1.031** |
| **CCND1** | **1.6588** | **-2.9485** | **1.7109** |
| **CCND2** | **1.6449** | **-1.1173** | **2.5562** |
| **CCND3** | **-1.0367** | **-2.2974** | **-4.519** |
| **CCNE1** | **1.1551** | **-1.9725** | **-2.4726** |
| **CCNF** | **1.3817** | **-1.9053** | **1.6162** |
| **CCNG1** | **1.108** | **1.0718** | **1.0673** |
| **CCNG2** | **-1.2075** | **3.4822** | **2.2564** |
| **CCNH** | **1.0267** | **-2.2658** | **-1.4006** |
| **CCNT1** | **1.1392** | **-1.1728** | **-2.2439** |
| **CDC16** | **1.0267** | **-1.5369** | **1.1204** |
| **CDC20** | **-4.1182** | **-3.7581** | **-6.0043** |
| **CDC25A** | **1.1551** | **-2.2815** | **-5.374** |
| **CDC25C** | **-1.7679** | **-1.3195** | **-2.0505** |
| **CDC34** | **-1.3031** | **-1.3195** | **-2.9608** |
| **CDC6** | **1.3086** | **-1.6358** | **-1.654** |
| **CDK1** | **-1.2414** | **-2.8879** | **-2.4897** |
| **CDK2** | **1.2553** | **-1.2397** | **-2.0083** |
| **CDK4** | **1.1392** | **1.2834** | **1.1282** |
| **CDK5R1** | **-1.2588** | **-2** | **-2.7435** |
| **CDK5RAP1** | **-1.3967** | **-1.3566** | **-2.4217** |
| **CDK6** | **-1.2329** | **-2.0994** | **-4.3954** |
| **CDK7** | **-1.1745** | **-1.6021** | **-1.0253** |
| **CDK8** | **-1.0439** | **-1.9862** | **-1.4804** |
| **CDKN1A** | **1.3086** | **-1.5692** | **1.314** |
| **CDKN1B** | **-1.2075** | **1.5911** | **-2.4726** |
| **CDKN2A** | **1.0338** | **-1.9725** | **-1.0837** |
| **CDKN2B** | **1.1631** | **-1.4439** | **-1.0614** |
| **CDKN3** | **-1.6958** | **-3.1602** | **-2.7245** |
| **CHEK1** | **1.1004** | **-1.6021** | **-1.6088** |
| **CHEK2** | **-1.0367** | **1.1567** | **-1.1065** |
| **CKS1B** | **-1.0014** | **-2.0139** | **-1.5648** |
| **CKS2** | **-2.6611** | **-3.9449** | **-3.1733** |
| **CUL1** | **-1.0154** | **-1.4142** | **-1.522** |
| **CUL2** | **1.0196** | **-2.7321** | **-1.2535** |
| **CUL3** | **-1.0439** | **-1.9453** | **-1.3159** |
| **E2F1** | **1.1235** | **-1.1892** | **-2.6317** |
| **E2F4** | **-1.0585** | **-2.0139** | **-4.1011** |
| **GADD45A** | **1.0483** | **-4.2871** | **-3.4486** |
| **GTSE1** | **-1.9616** | **-1.6133** | **-2.9814** |
| **HUS1** | **1.2209** | **-1.3379** | **-2.2284** |
| **KNTC1** | **1.0777** | **1.0867** | **1.7219** |
| **KPNA2** | **-2.8919** | **-3.0314** | **-5.5635** |
| **MAD2L1** | **-1.1111** | **-2.2501** | **-1.4907** |
| **MAD2L2** | **1.1392** | **-1.3379** | **-1.2449** |
| **MCM2** | **1.0629** | **1.1251** | **-1.1065** |
| **MCM3** | **1.2728** | **-1.2226** | **1.031** |
| **MCM4** | **-1.6495** | **1.2058** | **-4.4568** |
| **MCM5** | **1.041** | **-1.2397** | **-1.9399** |
| **MDM2** | **1.0703** | **-1.2483** | **-2.65** |
| **MKI67** | **-1.6155** | **-1.879** | **-3.1514** |
| **MNAT1** | **-1.0295** | **-2.639** | **-1.3717** |
| **MRE11A** | **1.0703** | **1.0718** | **1.0238** |
| **NBN** | **-1.1583** | **-2.3134** | **-2.2595** |
| **RAD1** | **1.0928** | **-2.0562** | **-1.654** |
| **RAD17** | **-1.0733** | **-1.5692** | **-1.2535** |
| **RAD51** | **1.3268** | **-1.5052** | **-1.3528** |
| **RAD9A** | **1.2906** | **-1.2226** | **-2.2752** |
| **RB1** | **-1.0367** | **1.4044** | **1.2693** |
| **RBBP8** | **1.1551** | **-2.0705** | **1.0454** |
| **RBL1** | **1.408** | **-3.1408** | **1.6771** |
| **RBL2** | **-1.1188** | **1.2483** | **-1.0614** |
| **SERTAD1** | **1.1004** | **1.0792** | **-1.62** |
| **SKP2** | **1.0629** | **-1.014** | **-1.9534** |
| **STMN1** | **-1.0658** | **-2.0705** | **-1.5648** |
| **TFDP1** | **1.2294** | **1.0867** | **-1.9265** |
| **TFDP2** | **-1.0224** | **-1.1892** | **-1.325** |
| **TP53** | **-1.0585** | **1.007** | **-2.291** |
| **WEE1** | **-1.3585** | **-1.2924** | **-1.9** |
